# Pharmacokinetic comparison between quercetin and quercetin 3-*O*-*β*-glucuronide in rats by UHPLC-MS/MS

**DOI:** 10.1038/srep35460

**Published:** 2016-10-24

**Authors:** Le-Le Yang, Na Xiao, Xiao-Wei Li, Yong Fan, Raphael N. Alolga, Xiao-Yue Sun, Shi-Lei Wang, Ping Li, Lian-Wen Qi

**Affiliations:** 1 State Key Laboratory of Natural Medicines, China Pharmaceutical University, No. 24 Tongjia Lane, Nanjing 210009, China

## Abstract

Quercetin is a natural flavonoid widely distributed in human diet and functional foods. Quercetin 3-*O*-*β*-glucuronide (Q3G) is present in wine and some medicinal plants. Quercetin and Q3G may be metabolized from each other *in vivo*. While quercetin has been the subject of many studies, the pharmacokinetic profiles of quercetin and Q3G (in animals) have not yet been compared. Herein, we prepared a column-based method for rapid isolation of Q3G from *Nelumbo nucifera*. Then, we developed an UHPLC-MS/MS method to compare the pharmacokinetics of quercetin and Q3G. Our results showed that the plasma concentration-time curves of quercetin and Q3G show two maxima (*T*_*max*1_ ≈ 0.75 h, *T*_*max*2_ ≈ 5 h). After oral administration of 100 mg/kg quercetin or 100 mg/kg Q3G in rats, predominantly Q3G was detected in plasma with *AUC* at 39529.2 ± 6108.2 mg·h·L^−1^ or 24625.1 ± 1563.8 mg·h·L^−1^, 18-fold higher than quercetin with *AUC* at 1583.9 ± 583.3 mg·h·L^−1^ or 1394.6 ± 868.1 mg·h·L^−1^, respectively. After intravenous injection of 10 mg/kg in rats, Q3G showed extensive tissue uptake in kidney (409.2 ± 118.4 ng/g), liver (166.1 ± 52.9 ng/g), heart (97.7 ± 22.6 ng/g), and brain (5.8 ± 1.2 ng/g). In conclusion, we have shown that Q3G is a major active component in plasma and tissue for oral administration of quercetin or Q3G.

Quercetin is one of the world’s most widely used dietary flavonoids and is one of the most extensively studied natural products nowadays^1^. It is widely distributed in the plant kingdom and abundant in apple, onion and tea^2^,^3^. As estimated, the average daily uptake of quercetin varies between 5 mg and 40 mg, even up to 200–500 mg^4^. Due to its medicinal effects, quercetin has always been an attractive natural product to study and understand. Quercetin 3-*O*-*β*-glucuronide (Q3G), a glucuronide conjugate of quercetin, is present in wine^5^, and in medicinal plants like *Hypericum hirsutum*^6^, *Nelumbo nucifera*^7^ and green beans^8^. Compared to the long history of use and widespread research on quercetin, the study of Q3G is much less extensive.

Pharmacokinetic features of drugs are important to understand their *in vivo* behavior and mechanisms of action^9^. Flavonoids are commonly taken orally and may be biotransformed by intestinal microbiota and metabolized in the liver^10^,^11^,^12^,^13^,^14^. When this happens, major metabolites present in plasma, not the parent compound, are bioactive. Metabolites may produce similar, stronger, or weaker effects compared with parent compounds in different models^15^,^16^,^17^. Quercetin and Q3G may be metabolized from one to each other *in vivo*^18^,^19^. It has been shown that Q3G was an effective anti-oxidative metabolite in rat plasma after oral administration of quercetin^20^. In previous studies, Q3G-containing metabolites accumulated in the aorta after administration of quercetin-rich food^21^. Notably, Q3G has been identified as the principal circulating form detected in human blood ranging from 0.1–10 μM^22^. After oral administration of Q3G, however, it remains unclear which can be absorbed into the blood circulation.

It is still unclear how the pharmacokinetic profile of Q3G compares to that of quercetin. In this study, a column-based method was developed for rapid preparation of Q3G from *Nelumbo nucifera*. We then established a sensitive and reliable UHPLC-MS/MS method for simultaneous determination of quercetin and Q3G in plasma. Subsequently, we employed the method to evaluate the pharmacokinetic differences after oral administration of quercetin and Q3G separately to rats. We also assessed the tissue disposition of Q3G after intravenous injection. The pharmacokinetics and tissue distribution results obtained from our study would be helpful to better understand the pharmacological actions of quercetin and Q3G.

## Results and Discussion

### Preparation, purification and spectral identification of Q3G

**Figure 1:**
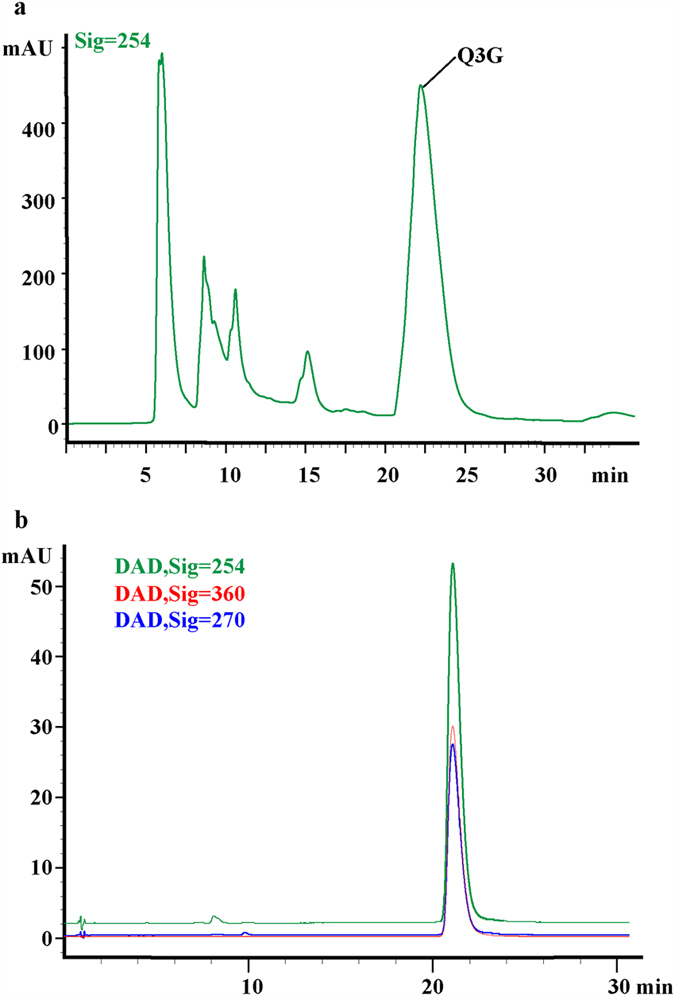
HPLC chromatogram of flavonoids from the *Nelumbo nucifera* extract recorded at 270 nm (**a**). The DAD-spectrum for determination the purity of quercetin 3-*O*-*β*-glucuronide (Q3G) recorded at 254, 270 and 360 nm (**b**).

The pharmacokinetics and tissue distribution of Q3G is limited. We found that Q3G was the dominant flavonoid of *Nelumbo nucifera*, which was consistent with the report by Chen *et al*.^23^,^24^. The purification of Q3G based on reversed phase HPLC with a mobile phase consisting of acetonitrile and water permitted the isolation of the pure compound (Fig. 1a). The purity of Q3G was determined by HPLC (Fig. 1b), and the structure was further confirmed by MS, NMR, HMBC and HSQC. This information has been included as additional Supplementary information (Supplementary Fig. S1). Using the separation system, we could prepare the standard of Q3G at the milligram level per HPLC run, which was sufficient for pharmacokinetics research in rats.

### Ultra-performance liquid chromatography and mass spectrometry

**Figure 2:**
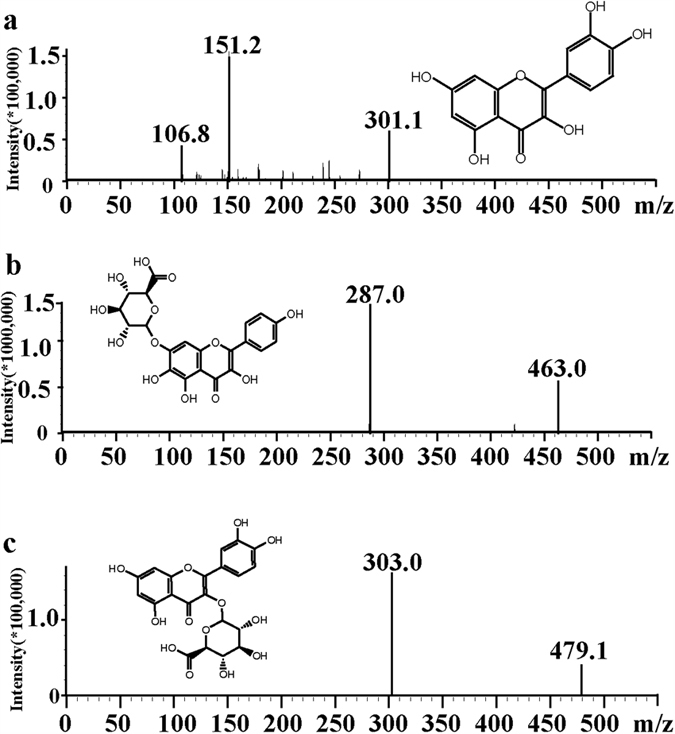
Chemical structures and mass spectra of quercetin (**a**), scutellarin (IS) (**b**), and quercetin-3-*O*-*β*-glucuronide (**c**). The mass spectrometric detection was conducted on a Shimadzu LCMS8050 triple-quadrupole mass spectrometer equipped with an ESI source in product ion scan mode.

The automatic tuning mode was performed to optimize MS parameters for the analyte and scutellarin (IS). Both positive and negative ion modes were examined, and the results showed that Q3G and IS had higher responses in the positive ion mode, while quercetin gave lower noise in negative ion mode. As shown in Fig. 2, the optimum multiple reaction monitoring (MRM) transition *m*/*z* 479.1 → 303.0 was chosen to quantify Q3G. Using similar conditions, the optimum MRM transition of quercetin was selected as *m*/*z* 301.1 → 151.2, and *m*/*z* 463.0 → 287.0 was selected for IS, respectively.

Although liquid–liquid extraction is one of the conventional methods for sample preparation, relatively time-consuming operation limits its high throughput analysis of biological samples. Consequently, we utilized the protein precipitation method as an advantageous sample purification technique for the extraction. As a result, protein precipitation with methanol provided a higher and similar recovery for both Q3G, quercetin and IS.

### Method validation

**Figure 3:**
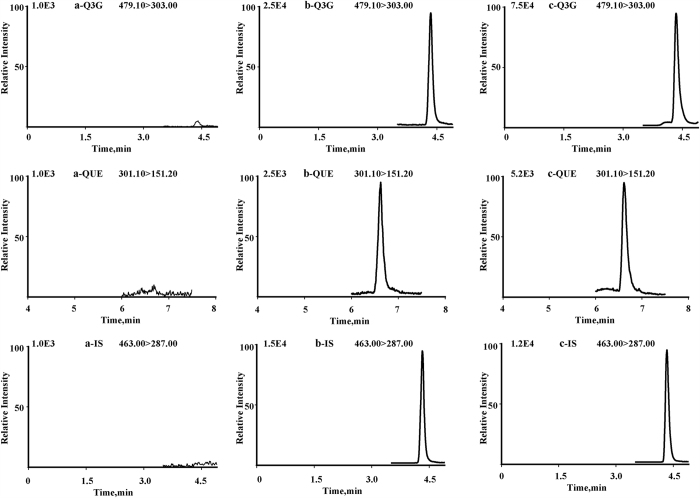
Representative multiple reaction monitoring (MRM) chromatograms of quercetin-3-*O-β*-glucuronide (Q3G), quercetin (QUE), and scutellarin (IS) in blank plasma (**a**), plasma spiked with Q3G at LLQQ and IS (**b**), plasma sample (**c**).

The outcome of the validation procedure outlined in the Method section is herein presented. *Selectivity*. Representative MRM chromatograms of the blank plasma, blank plasma spiked with the Q3G and IS, and plasma sample at 6 h after oral administration of 100 mg/kg Q3G are shown in Fig. 3. No endogenous interfering peak was observed in the plasma or tissue sample at the measured mass transitions and retention times of the analytes and IS for the highly selective MRM mode.

#### Calibration curves and lower limits of quantification

The calibration curves showed a correlation coefficient (r^2^) of 0.995 or better. The back calculated values of the calibration standards were within ±15% of their standard concentrations. The calibration curves, correlation coefficients and linearity range of quercetin and Q3G and quercetin in plasma and each tissue are listed in Supplementary Table S1. The lower limit of quantification (LLOQ) was 0.2 ng/ml with acceptable precision (relative standard deviation, RSD ≤ 20%) and accuracy (relative error, RE within ±20%), which were satisfactory for the pharmacokinetics and tissue distribution study of quercetin and Q3G.

#### Precision and accuracy

The determined concentrations of quercetin and Q3G in plasma and tissue samples at three quality control (QC) levels (2.5, 15, 150, or 800 ng/ml) and the results of precision and accuracy for intra- and inter-day are presented in Supplementary Table S2. The RSD of each QC concentration examined was measured to be within 9.5%, while the RE in the accuracy was less than 8.5%. These results showed that the precision and accuracy values were within the acceptable criteria of analysis.

#### Extraction recovery and matrix effect

In Supplementary Table S3, the extraction recoveries of quercetin and Q3G in plasma and tissues were similar, indicating that the extraction method is reliable. The matrix effect of quercetin and Q3G ranged from 91% to 111%, suggesting that no obvious co-eluting endogenous matrix influenced the ionization of Q3G, quercetin, and IS in the plasma or tissue samples.

#### Stability

As shown in Supplementary Table S4, quercetin and Q3G were stable under the conditions that the samples may experience since the bias in concentration was within ±15% of nominal values, and the established method was suitable for large scale sample analysis.

#### Carry-over and dilution integrity

No significant eluting peaks were observed after the injection of the highest calibration standard sample in the developed method. The accuracy of the diluted QC samples was within ±4.5% and the precision was less than 3.6%, which could satisfy the demands of dilution analysis.

### Pharmacokinetic study

**Figure 4:**
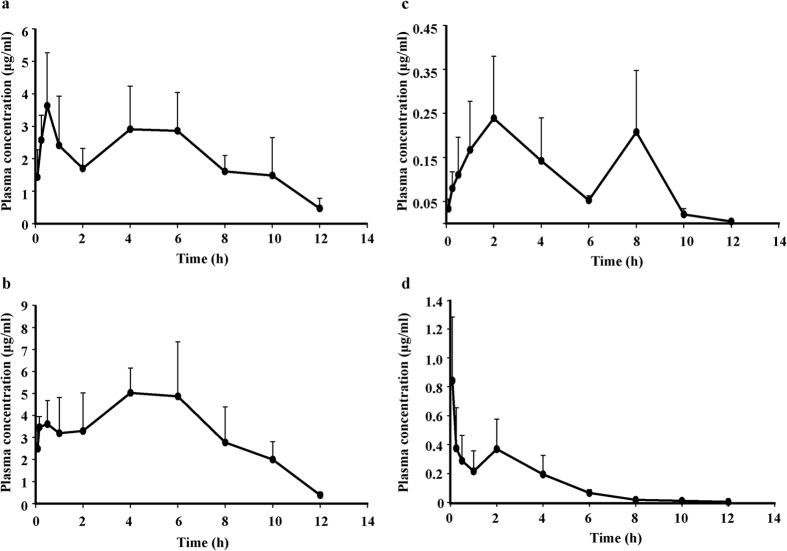
Mean plasma concentration-time curve of quercetin-3-*O*-*β*-glucuronide (Q3G) after oral administration of 100 mg/kg (**a**) quercetiin (QUE) (**b**) or QUE respectively in rats. (n = 5, mean ± SD). Mean plasma concentration-time profile of Q3G after oral administration of 100 mg/kg Q3G (**c**) or 100 mg/kg QUE (**d**) respectively in rats. (n = 5, mean ± SD).

The present analytical method was employed to study the pharmacokinetics of quercetin and Q3G in SD rats. The mean plasma concentration-time profile of quercetin and Q3G after intragastric administration (100 mg/kg dose) in rats is summarized in Fig. 4. Q3G showed more delayed absorption than quercetin aglycone, as the glucoside/glucuronide forms of quercetin are too polar to cross cellular membranes by diffusion, hampering their absorption and bioavailabilities. Interestingly, there existed a phenomenon of double peaks after orally administering quercetin or Q3G in rats, consistent with a previous research^25^. Research indicates that double-peaked pharmacokinetics may be caused by enterohepatic recirculation, delayed gastric emptying, and variability of absorption, which lead to relatively higher bioavailability^26^,^27^,^28^. Although several pharmacokinetic studies of quercetin and Q3G *in vivo* have been conducted, few studies have compared the metabolic profiles of quercetin and Q3G in animals. Morand, C. *et al*.^29^ demonstrated that 3-O-glucosylation increases the absorption of quercetin, while the bonding of a rhamnose to the aglycone significantly inhibits it. Shu, L. Y. *et al*.^30^ demonstrated that long-term exposure leads to a wide distribution of quercetin and quercetin metabolites in rat. However, the profiles of quercetin metabolites were not shown in these studies. In this study, we compared the levels and the profiles of quercetin and its major metabolite Q3G in SD rats.

The pharmacokinetic parameters of quercetin and Q3G were calculated by DAS software using the non-compartmental model and the results are presented in Table 1. Much more easily and faster absorption of Q3G with *C*_*max*_ at 6694.5 ± 1690 ng/ml or 4964.8 ± 1095.2 and quercetin with *C*_*max*_ at 842.1 ± 508.4 ng/ml or 316.1 ± 132 ng/ml were observed after oral administration of quercetin in contrast to that of Q3G. The corresponding plasma concentration and area under the curve (*AUC*) of quercetin was higher than that of Q3G for both plasma quercetin and Q3G. The mean *AUC* of plasma Q3G was statistically significantly different for the two compounds at same dose (*p* = *0.001*). The elimination half-life, *T*_*1/2*_, oral clearance (*CL*) and *T*_*max*_of Q3G for the two flavonoids were similar. These findings may be explained by the hydroxylation of Q3G into quercetin aglycone by luminal lactase phlorizin hydrolase or by intestinal microflora before absorption into blood. It is well known that flavonoid glycosides/glucuronides are barely absorbed from the small intestine due to their high hydrophilicity. Generally, flavonoid conjugates are hydrolyzed to aglycone by enterobacteria before passing through the small intestine, while flavonoid aglycone can be directly absorbed into epithelial cells in the intestine^31^,^32^. Based on the above absorption mechanism, the elimination half-life, *T*_*1/2*_, clearance, *CL* and *T*_*max*_ of quercetin for Q3G were higher and longer than that of quercetin, leading to the relative slow elimination of Q3G.

### Tissue distribution study

**Figure 5:**
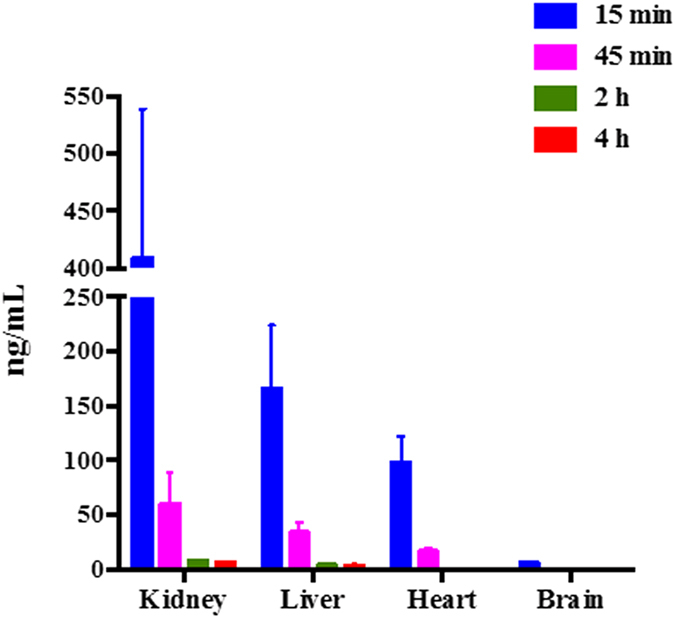
Distribution of quercetin-3-*O*-*β*-glucuronide (Q3G) in various tissues at different times after intravenous injection of 10 mg/kg Q3G.

The concentrations of Q3G in various tissue samples (kidney, liver, heart and brain) at 0.25 h, 0.75 h, 2 h and 4 h after intravenous administration of 10 mg/kg Q3G are listed in Fig. 5. The results showed that Q3G underwent a rapid and high degree of tissue distribution, compared with the profile of Q3G in plasma. Q3G was primarily present in organs supplied with abundant blood such as kidney, liver and heart. Q3G was distributed mainly in liver, and it showed the highest concentration (409.2 ± 118.4 ng/g) at 0.25 h after intravenous administration, followed by kidney (166.1 ± 52.9 ng/g), heart (97.7 ± 22.6 ng/g) and brain (5.8 ± 1.2 ng/g).The higher concentration in the kidney and liver, indicated that it is mainly metabolized and excreted in those tissues. At 0.75 h, nearly 85% of Q3G was cleared in rat tissues and plasma, indicating there was no accumulation of Q3G. A previous study demonstrated that accumulations of Q3G in the brain, and the brain-targeted Q3G has a strong inhibitory effect on Aβ aggregation^33^. Similarly, in the current analysis, Q3G was also detected and quantified in the brain, indicating that Q3G could penetrate the blood-brain barrier into the brain. Findings by Kawai *et al*.^34^ also demonstrated the localization and anti-inflammatory activity of Q3G in the brain.

## Conclusion

We showed that Q3G is a major bioactive component in plasma and tissues after oral administration of quercetin or Q3G. A simple and sensitive UPLC-MS/MS method was developed, validated and successfully used to compare the pharmacokinetics of Q3G with quercetin and investigate tissue distribution of Q3G. A simple one-step protein precipitation procedure for sample preparation in this study was adopted and the method employed only 25 μl of plasma. In addition, this method has a high sensitivity with LLOQ of 0.2 ng/ml, short analytical time of less than 10 min, good recovery with negligible matrix effect and similar recovery. To the best of our knowledge, this is the first report on the studies of the pharmacokinetics and tissue distribution of Q3G in rats. Furthermore, we compared the pharmacokinetics of quercetin with that of Q3G. The acquired data would be helpful for understanding the pharmacological effects of quercetin and Q3G.

## Methods

### Chemicals and materials

Q3G (purity ≥ 96.0%) was obtained from our lab. Scutellarin and quercetin (purity ≥ 98%) was purchased from Chengmust Biological Technology Co.,Ltd (Sichuan, China). The chemical structures of three compounds are shown in Fig. 2. HPLC grade acetonitrile and formic acid were purchased from Merck (Darmstadt, Germany). Water was purified by employing a Milli-Q water purification system (Millipore, France).

### Preparation of Q3G

Dried *Nelumbo nucifera* leaves were purchased from a local pharmacy. Extraction of Q3G from the leaves was performed according to the method stipulated by Ohara *et al*.^35^ with modifications. Briefly, the dried leaves (100 g) were extracted with 2 L of 50% aqueous ethanol at room temperature for 12 h. The extract was filtered, and then concentrated under reduced pressure. The concentrate was lyophilized, redissolved and then fractionated by HPLC apparatus (Shimadzu, Prominence LC-20AP, Japan) consisting of the following: A LC pump equipped with a LC-20 UV/Vis detector and a LC-8A column holder using YMC-Pack ODS-A columns (250 mm × 20 mm, S-5 μm, 12 nm). The mobile phase included 25% acetonitrile and Ultra-pure water containing 0.1% formic acid.

### MS, NMR, HMBC and HSQC spectral analysis of Q3G

The purity of Q3G was checked by Agilent 1260 HPLC–DAD on a Zorbax Eclipse Plus C18 column (2.1 × 150 mm, 3.5 μm). Elution conditions were as follows: 0.4 ml/min flow rate; 35 °C; solvent A, water/formic acid (99.9: 0.1 *v*/*v*); solvent B, acetonitrile (100%); isocratic elution of 11% B for 30 min, followed by washing and re-equilibration of the column. Qualitative analysis of Q3G was performed by an Agilent 6530 Q-TOF/MS system (Agilent Technologies, USA) equipped with an electrospray ionization (ESI) source. ^1^H, ^13^C-NMR, HMBC and HSQC data of Q3G (TMS as internal standard) were recorded in CD3OD on a Bruker AM-300 spectrometer at 300 MHz.

### Instrumentation and LC–MS/MS method

The triple quadrupole LC-MS/MS system consisted of a Shimadzu LC-30A chromatographic system and an ESI source-mass spectrometer (LCMS8050, Shimadzu, Japan). The system control and data analyses were performed by LabSolutions software (the software version: Version 5.65). Chromatographic separation was carried out on an Agilent ZORBAX SB-C18 column (150 mm × 4.6 mm, 5 μm) with a guard column (Agilent, USA). The HPLC was operated with a gradient mobile phase system consisting of water containing 0.1% formic acid (phase A) and acetonitrile (phase B) at a flow rate of 0.5 ml/min. The pump was programmed as follows: phase B was increased from 20% to 35% within the first 1.5 min, increased to 50% within the next 3 min, and from 50% to 70% within the next 1.5 min, then back to 20% (total gradient time: 10 min). A 5 μl sample was injected into the system with the auto-sampler conditioned at 4 °C and column temperature maintained at 35 °C. The mass spectrometer was operated in a positive and negative ion mode. MRM transitions were performed at m/z 479.1 → 303.0 for quercetin 3-G, m/z 463.0 → 287.0 for IS, and 301.1 → 151.2 for quercetin. Optimized values for Q1 Pre Bias, collision energy (CE), and Q3 Pre Bias were −24 V, −17 V, −21 V and 15 V for Q3G, −17 V, −24 V, −30 V for IS, and 20 V, 21 V, and 29 V for QC, respectively. The MS parameters were as follows: Nebulizing Gas Flow of 3 l/min; Heating Gas Flow of 10 l/min; Interface Temperature of 350 °C; DL Temperature of 200 °C; Heat Block Temperature of 400 °C; Drying Flow of 10 l/min. Representative product ion mass spectra of these compounds are exhibited in Fig. 2.

### Standard solutions, calibration and quality control samples

Stock solutions of 1.0 mg/ml of Q3G, quercetin and IS were prepared in 50% methanol. Subsequently, the working standard solutions of each analyte were prepared by serial dilution of the stock solution with 50% methanol to obtain the concentrations of 1–1000 ng/ml. All of the solutions were stored at −20 °C and brought to room temperature before use.

The calibration standards were prepared by spiking 25 μl blank plasma or tissue homogenate with 2.5 μl different concentrations of the working standard solutions, 2.5 μl IS solution on the day of analysis. Calibration curves of each drug were constructed in the range 1–1000 ng/mL for plasma (1, 2.5, 5, 10, 20, 40, 80, 160, 200, 500, 1000 ng/ml). QC samples (low QC, med QC and high QC) were prepared by the similar method as that for the calibration plots at concentrations of 2.5, 15, and 150 for tissue samples, 2.5,150 and 800 ng/ml for plasma). The standard working solutions and QC samples were stored at −20 °C.

### Sample preparation

An aliquot of rat plasma or tissue homogenate sample (25 μl) with 2.5 μl IS solution (scutellarin 100 ng/ml) was placed in a 1.5 ml Eppendorf tube and vortex-mixed for 3 min. The mixture was extracted with methanol (75 μl) by vortexing and then centrifuged at 13,000 rpm for 10 min at 4 °C. A volume of 5 μl of the supernatant was injected into the UPLC-MS/MS system for analysis.

### Method validation

The UPLC-MS/MS method employed in this work was validated in accordance with the FDA guidance for Bioanalytical Method^36^ with regard to specificity, linearity, precision and accuracy, extraction recovery and matrix effects, stability, carry-over effects and dilution integrity.

#### Specificity

The specificity of the method was investigated by analyzing six blank rat plasma samples, blank plasma spiked with Q3G, quercetin and IS, and actual rat plasma samples after administration of quercetin and Q3G, respectively.

#### Linearity and lower limit of quantification

Calibration curves were constructed by plotting the peak area ratios of the analytes to IS (Y-axis) against the nominal concentration of Q3G or quercetin (X-axis). This was then assessed by weighted least-squares linear regression analysis with a weighting factor (1/x^2^). LLOQ was defined as the lowest concentration with acceptable precision within 20% and accuracy of 80–120%.

#### Precision and accuracy

Intra-day and inter-day precision and accuracy were estimated by analyzing the three different QC concentrations for plasma and tissue in three replicates within a day and within three days. Acceptable accuracy (RE) and precision (RSD) values were set within ±15% of the nominal concentration, except LLOQ (within ±20%).

#### Extraction recovery and Matrix effect

Extraction recovery of quercetin and Q3G in plasma and tissue samples were evaluated by comparing the peak area of the extracted samples with those of the corresponding spiked standard solutions (n = 6). For matrix effect evaluation, the peak areas of analytes spiked into blank plasma and tissue were compared with those dissolved in 50% methanol solution at three QC concentrations after extraction.

#### Dilution integrity

This was done to assess the extent to which the upper concentration limit of this method can be extended with acceptable precision and accuracy. Six replicate QC samples were processed in blank rat plasma at a concentration of about 2 times of the uppermost calibration standard, then diluted 10 fold with additional screened blank plasma before analysis.

#### Stability

The stabilities of analytes in rat plasma or tissue were evaluated under various storage and process conditions. Short-term stability was evaluated after the exposure of QC samples to room temperature for 6 h. For post-preparative stability, the QC samples were kept in the auto-sampler (24 h, 4 °C) prior to analysis. Freeze/thaw stability was evaluated by analyzing QC samples at three levels after undergoing three freeze (−80 °C)-thaw (ambient) cycles.

### Pharmacokinetic and tissue distribution study

Thirty-four Sprague-Dawley (SD) male rats (250–300 g, 10–12 weeks) were obtained from the Sino-British SIPPR/BK Lab Animal Ltd. (Shanghai, China). These rats were kept in a controlled room with temperature of (22 ± 2) °C and a relative humidity of (50 ± 10) % for one week prior to the start of the experiments. All rats were provided with food and water and fasted overnight before starting the experiment with access to water. All experiments and animal care were conducted in accordance with the Provision and General Recommendation of Chinese Experimental Animals Administration Legislation and were approved by the Science and Technology Department of Jiangsu Province (license number: SYXK (Su) 2012–0005).

For the pharmacokinetic study, after oral administration of 100 mg/kg Q3G and 100 mg/kg quercetin to 10 rats (n = 5), approximately 200 μl blood was collected from the jugular vein cannula into heparinized tubes at 0 (pre-drug), 0.083 h, 0.25 h, 0.5 h, 1 h, 2 h, 4 h, 6 h, 8 h, 10 h, 12 h and 24 h post-dose. The whole blood samples were immediately centrifuged at 3,000 rpm for 10 min, and stored at –80 °C until analysis.

For tissue distribution study, 24 rats were randomly assigned to four groups (6 rats/group), and given 10 mg/kg Q3G by intravenous administration. The vital organs of interest were collected immediately after the rats were euthanized at 0.5 h, 1 h, 2 h and 4 h and weighed accurately. The tissues were washed in normal saline and blotted dry with filter paper. An accurately weighed amount of each tissue sample (0.5 g) was homogenized in normal saline (1 ml) and stored at −80 °C until analysis.

### Data analysis

The pharmacokinetic parameters were calculated by the DAS Software (version 2.0, China State Drug Administration) using non-compartmental model. Significant differences between group values were analyzed using a one-tailed Student’s t-test. Differences were considered statistically significant at P-values less than 0.05.

## Additional Information

**How to cite this article**: Yang, L.-L. *et al*. Pharmacokinetic comparison between quercetin and quercetin 3-*O*-*β*-glucuronide in rats by UHPLC-MS/MS. *Sci. Rep.* **6**, 35460; doi: 10.1038/srep35460 (2016).

## Supplementary Material

Supplementary Information

## Figures and Tables

**Table 1:** Pharmacokinetic parameters (mean ± SD) of quercetin-3-*O*-*β*-glucuronide (Q3G) and quercetin (QUE) after oral administration of 100 mg/kg Q3G or quercetin (n = 5).

Pharmacokinetic parameters	Unit	Value			
		Q3G		QUE	
		Q3G(oral)	QUE(oral)	Q3G(oral)	QUE(oral)
AUC(0–t)	mg·h·L^−1^	24625.1 ± 1563.8	39529.2 ± 6108.2	1394.6 ± 868.0	1583.9 ± 583.3
AUC(0–∞)	mg·h·L^−1^	31328.4 ± 10693.8	42730.3 ± 7511.1	1400.7 ± 866.8	1597.3 ± 589.7
t_1/2_	h	2.6 ± 0.9	2.4 ± 0.8	1.1 ± 0.6	0.8
T_max_	h	3.1 ± 2.3	5.0 ± 1.2	2.9 ± 3.5	0.3 ± 0.1
CL	L/h/kg	0.003 ± 0.001	0.002 ± 0.001	0.4 ± 0.2	0.8 ± 0.3
C_max_	mg/L	4964.8 ± 1095.2	6694.5 ± 1690.0	316.1 ± 132.0	842.1 ± 508.4
