# Supplementary material for: Pharmacokinetic comparison between quercetin and quercetin 3-O-β-glucuronide in rats by UHPLC-MS/MS
Source: Sci Rep. 2016 Oct 24;6:35460. doi: 10.1038/srep35460 (PMC5075792; doi:10.1038/srep35460)
Supplement: Supplementary Information [file srep35460-s1.pdf]

Supplementary Information

Pharmacokinetic comparison between quercetin and quercetin 3-*O*-*β*-glucuronide in rats by UHPLC-MS/MS

Le-Le Yang, Na Xiao, Xiao-Wei Li, Yong Fan, Raphael N. Alolga, Xiao-Yue Sun, Shi-Lei Wang, Ping Li* & Lian-Wen Qi*

State Key Laboratory of Natural Medicines, China Pharmaceutical University, No. 24 Tongjia Lane, Nanjing 210009, China.

*Corresponding author:
Tel/Fax: +86-25 86185559
E-mail address: Qilw@cpu.edu.cn (L.-W. Qi), Liping2004@126.com (P. Li).

Supplementary Figure S1. The MS (a), structure (b), ^{1}H (c), ^{13}C-NMR (d), HMBC (e) and HSQC (f) data of quercetin 3-O-β-glucuronide (Q3G).

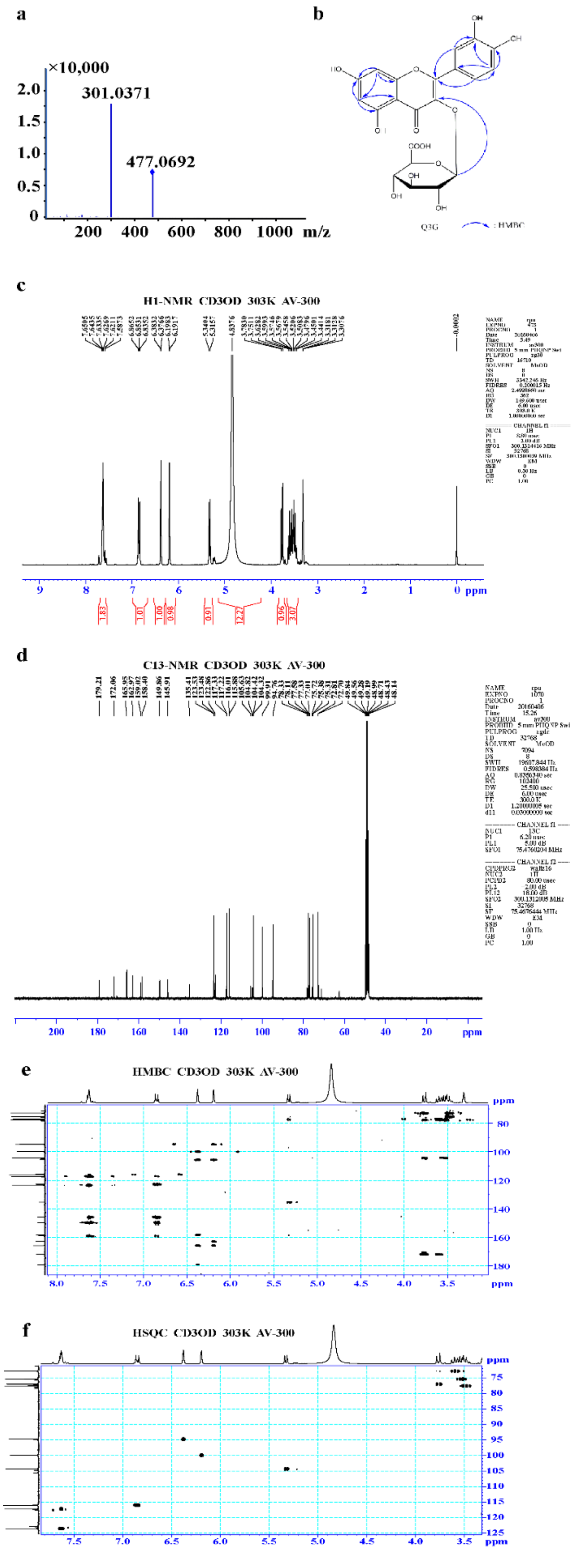

The ^{1}H NMR and ^{13}C NMR spectra of Q3G were as follows:

^{1}H NMR (300 MHz, C_3D_6O) δ 6.19 (1H, d, *J* = 1.98 Hz, H-6), 6.38 (1H, d, *J* = 1.98 Hz, H-8), 7.64 (1H, br, H-2'), 7.60 (1H, br, H-6'), 6.85 (1H, d, *J* = 9 Hz, H-5'), 5.33 (1H, d, *J* = 7.4 Hz, H-1''), 3.50 (1H, t, *J* = 8.6 Hz, H-2''), 3.54 (1H, t, *J* = 8.6 Hz, H-3''), 3.60 (1H, t, *J* = 8.7 Hz, H-4''), 3.76 (1H, d, *J* = 9.6 Hz, H-5'');

^{13}C NMR (75 MHz, C_3D_6O) δ: 159.02 (C-2), 135.41 (C-3), 179.21 (C-4), 162.97 (C-5), 99.91 (C-6), 165.95 (C-7), 94.76 (C-8), 158.40 (C-9), 105.63 (C-10), 122.86 (C-1'), 117.22 (C-2'), 145.91 (C-3'), 149.86 (C-4'), 116.01 (C-5'), 123.53 (C-6'), 104.32 (C-1''), 75.38 (C-2''), 77.58 (C-3''), 72.81 (C-4''), 77.01 (C-5''), 172.06 (C-6'').

The ^{13}C NMR spectrum showed six carbon signals (δ 162.97, 99.91, 165.95, 94.76,158.40 and 105.63), and the ^{1}H NMR spectrum showed two proton signals at δ 6.38 and 6.19 possessed the same coupling constant (*J* = 1.98 Hz), which could be assigned to A ring of quercetin with the HSQC spectrum. The ^{13}C NMR spectrum showed six carbon signals (δ 122.86, 117.22, 145.91, 149.86,116.01, and 123.53), and the ^{1}H NMR spectrum showed three proton signals at δ 7.64, 7.60, and 6.85, which could be assigned to B ring of quercetin with the HSQC spectrum. These data unambiguously supported the conclusion that the structure of the aglycone moiety of **Q3G** is quercetin.

The ^{13}C-NMR spectrum of **Q3G** showed six carbon signals assigned to a glucuronic acid moiety, including one anomeric carbon (δ 104.32) and one carboxyl carbon (δ 172.06), besides those of an aglycone moiety. In the ^{1}H-NMR spectrum of **Q3G**, a sequential *trans*-1,2-diaxial relationship of H-1''–H-5'' (δ, 5.32–3.50, *J* = 8.6–

9.6 Hz) indicated the presence of a β-D-glucuropyranosyl moiety in **Q3G**.

Moreover, the cross peak from H-1'' (*δ* 5.32) to C-3 (*δ* 135.41) in the HMBC spectrum indicated that the glucuronic acid was conjugated to the C-3 position of the quercetin. Therefore, it was concluded that the structure of **Q3G** was quercetin 3-*O*-*β*-*D*-glucuronide (Q3G).

Supplementary Table S1. Calibration curve, correlation coefficients and linear ranges of Q3G and quercetin in different bio-samples.

Compound	Bio-sample	Calibration curve	Correlation coefficient(r^2)	Linear range (ng/ml)
**Q3G**	Plasma	Y = 0.0453X + 0.0533	0.9968	5-1000
	Liver	Y = 0.0107X + 0.00277	0.9978	1-50
	Brain	Y = 0.0110X + 0.00240	0.9968	0.2-25
	Heart	Y = 0.0141X + 0.00521	0.9949	1-50
	Kidney	Y = 0.0141X + 0.00255	0.9996	1-100
**Quercetin**	Plasma	Y = 0.0260X + 0.00628	0.9979	1-200

Supplementary Table S2. Summary of the inter- and intra-day variability for the assay of Q3G and quercetin detected by UHPLC-MS/MS method in rat plasma and tissues (Mean ± SD, *n* = 5). R.S.D: relative standard deviation (%); R.E.: relative error (%).

Compound	Bio-sample	Normal Concentration (ng/ml)	Inter-day(n=5)			Inter-day(n=5)		
			Measured Concentration (ng/ml)	Precision R.S.D (%)	Accuracy R.E. (%)	Measured Concentration (ng/ml)	Precision R.S.D (%)	Accuracy R.E. (%)
**Q3G**	Plasma	15	15.2 ± 1.3	8.5	1.2	15.3 ± 0.8	5.5	2.1
		150	150 ± 7.3	4.9	-1.0	149.4 ±6.5	4.3	-0.4
		800	786.8±74.5	9.5	-1.7	774.1±60.7	7.8	-3.2
	Liver	2.5	2.50 ± 0.11	4.5	-0.02	2.44 ± 0.16	6.4	-2.2
		15	14.0 ± 0.59	4.2	-6.9	14.5 ± 1.08	7.4	-3.1
		25	24.6 ± 1.43	5.8	-1.5	24.9 ± 1.20	4.8	-0.3
	Brain	0.5	0.5 ± 0.03	5.6	4.8	0.5 ± 0.04	7.0	4.1
		2.5	2.3 ± 0.14	6.1	-6.1	2.5 ± 0.19	7.8	-1.5
		15	16.3 ± 0.51	3.1	8.5	15.9 ± 1.14	7.2	6.2
	Heart	2.5	2.6 ± 0.07	2.8	5.8	2.4 ± 0.21	8.7	-4.7
		15	15.0 ± 1.42	9.5	0.3	14.3 ± 1.01	7.1	-2.6
		25	25.0 ± 1.22	4.9	0.2	23.8 ± 1.96	8.2	-5.0
	Kidney	2.5	2.6 ± 0.09	3.5	2.1	2.5 ± 0.16	6.2	1.9
		15	15.7 ± 1.05	6.7	4.4	15.2 ± 0.99	6.5	1.4
		80	76.3 ± 0.69	0.9	-4.6	77.0 ± 1.57	2.0	-3.8
**Quercetin**	Plasma	2.5	2.4 ± 0.2	7.8	-2.1	2.4 ± 0.2	7.2	-5.7
		15	15.0 ± 1.3	8.6	-0.2	14.8 ± 1.1	7.1	-1.1
		150	150 ± 2.9	3.7	-3.2	150±4.1	5.4	-5.1

Supplementary Table S3. Matrix effect and extraction recovery of Q3G and quercetin.

Compound	Bio-sample	Normal Concentration (ng/ml)	Matrix effect (%)	R.S.D (%)	Extraction recovery (%)	R.S.D (%)
**Q3G**	Plasma	15	97.7±5.3	5.4	82.1±3.4	4.2
		150	102.3±6.2	6.1	75.8±2.1	2.8
		800	102.6±1.5	1.5	79.0±1.0	1.2
	Liver	2.5	99.5 ± 2.0	2.0	64.5 ± 1.9	2.9
		15	95.3 ± 5.3	5.6	67.3 ± 1.5	2.2
		25	92.6 ± 3.2	3.5	65.4 ± 2.1	3.2
	Brain	0.5	100.9 ± 7.3	7.2	70.6 ± 6.8	9.6
		2.5	90.6 ± 1.2	1.3	73.6 ± 7.5	10.2
		15	97.1 ± 9.3	9.5	68.6 ± 8.5	12.4
	Heart	2.5	108.5 ± 5.2	4.8	66.0 ± 2.2	3.3
		15	101.8 ± 4.1	4.1	70.7 ± 3.5	4.9
		25	96.1 ± 3.3	3.4	67.7 ± 4.4	6.5
	Kidney	2.5	106.1 ± 3.5	3.3	70.9 ± 2.4	3.4
		15	111.2 ± 10.9	9.8	67.9 ± 2.1	3.0
		80	99.3 ± 3.5	3.5	72.9 ± 0.8	1.0
**Quercetin**	Plasma	2.5	91.1±5.9	6.4	99.3±4.5	4.6
		15	91.6±3.2	3.5	104.4±9.0	8.6
		150	93.7±7.0	7.4	101.7±8.0	7.9

Supplementary Table S4. Stability of Q3G and quercetin in rat plasma and tissues during sample preparation and analytical process.

Compound	Bio-sample	Normal Concentration (ng/ml)	Auto-sampler (24 h, 4°C)		Three freeze/thaw cycles		Room temperature (6 h)	
			Precision R.S.D (%)	Accuracy R.E. (%)	Precision R.S.D (%)	Accuracy R.E. (%)	Precision R.S.D (%)	Accuracy R.E. (%)
**Q3G**	Plasma	15	3.6	3.7	4.3	2.7	4.0	-1.0
		150	2.1	-2.3	1.6	0.6	0.8	-4.8
		800	1.6	-3.1	2.4	-6.9	4.6	-1.2
	Liver	2.5	3.2	-2.6	4.4	-1.2	4.8	-2.1
		15	7.1	-8.2	6.0	-8.9	5.0	-2.0
		25	3.3	-5.8	2.8	3.7	3.3	5.9
	Brain	0.5	7.5	4.6	4.6	-0.4	4.3	-2.3
		2.5	8.8	-1.2	6.8	0.9	6.8	-2.2
		15	5.1	7.3	4.9	5.7	2.2	5.4
	Heart	2.5	3.8	-10.4	6.8	-9.0	1.1	-12.9
		15	9.1	-0.6	7.6	0.7	8.0	-2.7
		25	1.7	0.6	8.9	-0.8	6.4	-4.0
	Kidney	2.5	8.5	2.9	3.6	8.9	6.4	-1.5
		15	4.1	7.3	7.6	6.5	8.1	5.8
		80	0.9	-4.1	1.0	-4.1	1.7	-3.4
**Quercetin**	Plasma	2.5	6.9	-1.6	5.5	-4.7	3.1	-0.9
		15	9.9	-0.8	9.7	-2.2	4.5	-5.4
		150	5.5	0.9	1.6	4.5	4.0	0.0
